# Immune complexes containing scleroderma-specific autoantibodies induce a profibrotic and proinflammatory phenotype in skin fibroblasts

**DOI:** 10.1186/s13075-018-1689-6

**Published:** 2018-08-29

**Authors:** Elena Raschi, Cecilia Beatrice Chighizola, Laura Cesana, Daniela Privitera, Francesca Ingegnoli, Claudio Mastaglio, Pier Luigi Meroni, Maria Orietta Borghi

**Affiliations:** 1 Experimental Laboratory of Immunological and Rheumatologic Researches, IRCCS Istituto Auxologico Italiano, Via Zucchi 18, 20095 Cusano Milanino, Milan, Italy; 2 Department of Clinical Sciences and Community Health, University of Milan, Via Festa del Perdono 7, 20122 Milan, Italy; 3 Allergology, Clinical Immunology and Rheumatology Unit, IRCCS Istituto Auxologico Italiano, Piazzale Brescia 20, 20149 Milan, Italy; 4 Division of Rheumatology, ASST G. Pini, Piazza C Ferrari 1, 20122 Milan, Italy; 5 Rheumatology Unit, Ospedale Moriggia-Pelascini, Via Pelascini 3, 22015 Gravedona, Como Italy

**Keywords:** Systemic sclerosis, Autoantibodies, Immune complexes, Toll-like receptors, Fibroblasts, Fibrosis, Inflammation

## Abstract

**Background:**

In systemic sclerosis (SSc), autoantibodies provide the most accurate tool to predict the disease subset and pattern of organ involvement. Scleroderma autoantibodies target nucleic acids or DNA/RNA-binding proteins, thus SSc immune complexes (ICs) can embed nucleic acids. Our working hypothesis envisaged that ICs containing scleroderma-specific autoantibodies might elicit proinflammatory and profibrotic effects in skin fibroblasts.

**Methods:**

Fibroblasts were isolated from skin biopsies obtained from healthy subjects and patients with diffuse cutaneous SSc (dcSSc). ICs were purified by polyethylene-glycol precipitation from sera of SSc patients bearing different autoantibodies. ICs from patients with systemic lupus erythematosus (SLE) and primary anti-phospholipid syndrome (PAPS) and from normal healthy subjects (NHS) were used as controls. After incubation with ICs, fibroblasts were evaluated for ICAM-1 expression, interleukin (IL)-6, IL-8, monocyte chemoattractant protein (MCP)-1, matrix metalloproteinase (MMP)-2, tumor growth factor (TGF)-β1 and Pro-CollagenIα1 secretion, *collagen (col)Iα1*, *mmp-1*, *toll-like receptor (tlr)2*, *tlr3*, *tlr4*, *tlr7*, *tlr8*, *tlr9*, *interferon (ifn)-α*, *ifn-β* and *endothelin-1* mRNA, and NFκB, p38MAPK and SAPK-JNK activation rate. Experiments were also performed after pretreatment with DNase I/RNase and NFκB/p38MAPK inhibitors.

**Results:**

The antigenic reactivity for each SSc-IC mirrored the corresponding serum autoantibody specificity, while no positivity was observed in NHS-ICs or sera. SSc-ICs but not NHS-ICs increased ICAM-1 expression, stimulated IL-6, IL-8, MMP-2, MCP-1, TGF-β1 and Pro-CollagenIα1 secretion, upregulated *et-1*, *ifn-α*, *ifn-β*, *tlr2, tlr3* and *tlr4*, and activated NFκB, p38MAPK and SAPK-JNK. *tlr9* was significantly upregulated by ARA-ICs, *mmp-1* was significantly induced by ACA-ICs whereas *colIα1* was not modulated by any SSc-ICs. SLE-ICs and PAPS-ICs significantly upregulated MMP-2 and activated NFκB, p38MAPK and SAPK-JNK. SLE-ICs and PAPS-ICs did not affect *colIα1*, *mmp-1* and Pro-CollagenIα1. DNase I and RNase treatment significantly reduced the upregulation of study mediators induced by SSc-ICs. Pretreatment with NFκB/p38MAPK inhibitors suggested that response to anti-Th/To-ICs was preferentially mediated by p38MAPK whereas ATA-ICs, ACA-ICs and ARA-ICs engaged both mediators. In dcSSc fibroblasts, stimulation with SSc-ICs and NHS-ICs upregulated IL-6 and IL-8.

**Conclusions:**

These data provide the first demonstration of the proinflammatory and profibrotic effects of SSc-ICs on fibroblasts, suggesting the potential pathogenicity of SSc autoantibodies. These effects might be mediated by Toll-like receptors via the interaction with nucleic acid fragments embedded in SSc-ICs.

## Background

Systemic sclerosis (SSc) is a chronic systemic autoimmune disease characterized by three cardinal processes: fibrotic derangement of the skin and visceral organs, endothelial damage and immune activation [1]. A hallmark feature of SSc is the production of autoantibodies: anti-nuclear antibodies (ANA) are detectable in more than 95% of patients at diagnosis [2]. SSc-specific autoantibodies typically precede disease onset, implicating they are not a mere reflection of the disease process [3]. Importantly, they provide the most accurate tool to predict disease subsets and the pattern of organ complications [4, 5]. Noteworthy, IgG transfer from SSc patients in skin-humanized SCID mice induced significant dermal fibrosis [6]. Despite these in-vivo data, the precise diagnostic accuracy and the strong prognostic role played by scleroderma-specific autoantibodies, scarce in-vitro evidence has been raised in support of their pathogenic potential. To date, available studies have mainly focused on anti-DNA topoisomerase I antibodies (ATA), that were demonstrated to bind to fibroblasts via surface-bound topoisomerase I inducing adhesion and activation of co-cultured monocytes [7, 8]. Antibodies against centromeric proteins (ACA) were reported to prevent the transactivation of the epidermal growth factor receptor and the subsequent secretion of interleukin (IL)-8 [9].

Immune complexes (ICs) are formed upon interaction between autoantibodies and soluble target antigens. ICs contribute to the pathogenesis of several autoimmune diseases, such as systemic lupus erythematosus (SLE), Sjögren’s syndrome and rheumatoid arthritis [10–13]. Since the role of ICs in SSc has never been investigated, the aim of this study was to assess the proinflammatory and profibrotic effects of scleroderma ICs, using skin fibroblasts from healthy subjects as an in-vitro model. Since scleroderma autoantibodies engage nucleic acids or DNA/RNA-binding proteins as antigenic targets, SSc-ICs can embed nucleic acids [14]. The effects of SSc-ICs on target cells might thus be mediated by Toll-like receptors (TLRs) interacting with nucleic acid fragments. TLRs are expressed by many nonimmune cells, including fibroblasts, and are crucial in sensing pathogen-associated and damage-associated molecular patterns. In humans, 10 TLRs have been described: TLR2 and TLR4 are involved in the recognition of microbial molecules; TLR3 recognizes double-stranded RNA; TLR7 and TLR8 bind single-stranded RNA; and TLR9 engages single-stranded DNA [15].

As a whole, the data presented in this study provide the first demonstration of the proinflammatory and profibrotic effects of SSc-ICs on healthy skin fibroblasts. The evidence raised in this work suggests that such effects might be ascribed to nucleic acid components of SSc-ICs via the interaction with TLRs on target cells. As a whole, these data shed new light on the pathogenic role of scleroderma-associated autoantibodies, potentially broadening our understanding of SSc etiopathogenesis.

## Methods

### Serum samples

Serum samples were obtained from 16 patients with SSc fulfilling the 2013 ACR/EULAR criteria [16]. All patients had ANA upon indirect immunofluorescence on HEp-2 cells, at a titer greater than 1:160, with staining patterns consistent with the antigenic specificity. Five patients carried ATA, five ACA, three anti-RNA polymerase III antibodies (ARA) and three anti-Th/To antibodies (anti-Th/To). The remaining autoantibody profile was negative. In all cases, antibody reactivities against scleroderma antigens were confirmed using two different techniques: line blot (EUROLINE-SSc profile kit; Euroimmun, Lubeck, Germany) and chemiluminescent immunoassays (QUANTA Flash; INOVA Diagnostics, San Diego, CA, USA). Seven patients were diagnosed with diffuse cutaneous SSc (dcSSc), and the remaining subjects had limited cutaneous involvement [17]. All patients were female, the median age was 48 years and the median disease duration from the first non-Raynaud’s phenomenon symptom to blood withdrawal was 31 months. Two SLE patients were recruited: one patient carried anti-Sm, anti-U1 ribonucleoprotein (RNP) and anti-double stranded DNA antibodies; the other harbored anti-Sm [18]. Serum was also obtained from two subjects with primary anti-phospholipid syndrome (PAPS) and positive lupus anticoagulant test, anti-cardiolipin and anti-β2 glycoprotein I IgG antibodies [19]. Eight normal healthy subjects (NHS) with no autoimmune disease and negative autoantibody profile were enrolled. Serum samples were stored at − 20 °C.

### Healthy skin fibroblast cell culture

Dermal fibroblasts were isolated from skin biopsies from eight NHS. Under local anesthesia with 1% xylocaine, 5-mm punch skin biopsies were performed in the distal forearm. Samples were minced into small pieces, and digested by collagenase type I (ThermoFisher Scientific Inc., Waltham, MA, USA) for 2 h at 37 °C with 5% CO_2_. After centrifugation at 300 × *g* for 10 min, pellets were resuspended in 1 ml D-MEM (Gibco-Life Technologies, Groningen, the Netherlands) supplemented with 20% fetal bovine serum (FBS; PAA-GE Healthcare, Buckinghamshire, UK), 2 mM glutamine (Sigma-Aldrich, Saint Louis, MO, USA), penicillin (100 U/ml)–streptomycin (100 μg/ml) (Sigma-Aldrich) and transferred into a T25 plate (Corning Incorporated, NY, USA). Cultures were maintained at 37 °C in 5% CO_2_-humidified incubator until confluence. Nonadherent cells and dermal tissue were removed by washing, and established fibroblasts were passaged after trypsin/EDTA (ThermoFisher Scientific) release up to the eight passage. Cells were maintained in D-MEM with 10% FBS, 2 mM glutamine, penicillin (100 U/ml)–streptomycin (100 μg/ml) (ThermoFisher Scientific) or incubated overnight in D-MEM with 1% FBS before functional studies.

The purity of fibroblast culture was 98% as detected by flow cytometry using a mouse anti-human CD90 and a mouse anti-human CD45 antibodies–PE conjugated (BD Biosciences, San Jose, CA, USA).

### Immune complexes

ICs were precipitated from sera of NHS and patients. Briefly, serum samples were mixed with ice-cold 5% polyethylene-glycol (PEG) 6000 (Sigma-Aldrich)–0.1 M EDTA (Bioscience, Inc., La Jolla, CA, USA) and incubated overnight at 4 °C. Samples were diluted three times with 2.5% PEG 6000 in RPMI (Euroclone S.p.A., Pero, Italy), layered on top of 2.5% PEG 6000 supplemented with 5% human serum albumin (Sigma-Aldrich) and centrifuged at 2100 × *g* at 4 °C for 20 min. Pellets were dissolved in D-PBS to the initial serum volume and immediately used at 1:2 dilution [20].

The IC amount in PEG precipitates was quantified using Quanta Lite C1q CIC ELISA (INOVA Diagnostics), a sensitive and specific assay exploiting soluble IC binding to C1q [21, 22]. The presence of specific autoantibodies in PEG-precipitated ICs was tested using the commercial EUROLINE-SSc profile kit. The nucleic acid concentration (ng/μl) in IC preparations was evaluated by NanoPhotometer Pearl at 260 nm (Implen GmbH, München, Germany).

Every sample was used in triplicate, and each experiment was repeated twice using SSc-ICs isolated from all patients for each autoantibody specificity and control ICs.

The potential endotoxin contamination of IC preparations was ruled out by limulus amoebocyte lysate (LAL) gel-clot test (Pyrosate Kit, sensitivity 0.25 EU/ml; Associates of Cape Cod Incorporated, East Falmouth, MA, USA).

### ICAM-1 expression

ICAM-1 surface levels were evaluated by home-made cell ELISA, as in previous studies on HUVECs [23]. Confluent fibroblast monolayers were rested in D-MEM with 1% FBS overnight in a 96-well plate.

After 24-h incubation with 100 μl/well of SSc-ICs, NHS-ICs, LPS (1 μg/ml; R&D Systems, Minneapolis, MN, USA), poly(I:C) (1 μg/ml; Sigma-Aldrich) or medium alone, cells were washed twice with HBSS (Sigma-Aldrich) and incubated for 60 min at room temperature with 100 μl/well of murine monoclonal IgG specific for human ICAM-1 (CD54; R&D Systems). The antibody was used at a final dilution of 1:500 in HBSS-FBS 2.5%. After two additional washes, cells were incubated for 90 min at room temperature with 100 μl of phosphatase-conjugated goat anti-mouse IgG (Cappel, Cochranville, PA, USA). The secondary antibody was used at a dilution of 1:1000 in HBSS–FBS 10%. After two washes with HBSS, 100 μl of the enzymatic substrate (*p*-nitrophenylphosphate in 0.05 M Mg-carbonate buffer pH 9.8; Sigma-Aldrich) was added. The optical density (OD) values were evaluated at 405 nm after 30 min of incubation by a semiautomatic reader (Titertek Multiskan MCC/340; Titertek Instruments Inc., Pforzheim, Germany).

### IL-6, IL-8, matrix metalloproteinase-2, monocyte chemoattractant protein-1 and tumor growth factor protein secretion

IL-6, IL-8, matrix metalloproteinase (MMP)-2, monocyte chemoattractant protein (MCP)-1 and tumor growth factor (TGF)-β1 release was evaluated in culture supernatants after 48 h incubation with SSc-ICs, NHS-ICs or TLR synthetic agonists (LPS and poly(I:C)) by commercial ELISAs (R&D Systems).

### Pro-CollagenIα1 secretion

Pro-CollagenIα1 secretion in culture supernatants was evaluated after 24 h incubation with SSc-ICs, NHS-ICs or recombinant human TGF-β1 (10 ng/ml; PreproTech, Rocky Hill, JN, USA) by the human Pro-CollagenIα1 DuoSet ELISA Kit (R&D Systems).

### *tlr2*, *tlr3*, *tlr4*, *tlr7*, *tlr8*, *tlr9*, *interferon-α*, *interferon-β*, *endothelin-1*, *collagenIα1* and *mmp-1* mRNA expression levels

Total RNA from fibroblasts was purified using Trizol Reagent (ThermoFisher Scientific). Amplification Grade DNase I (ThermoFisher Scientific) was used to eliminate residual genomic DNA. A reverse transcription reaction was performed using the SuperScript™ First-Strand Synthesis System for RT-PCR (ThermoFisher Scientific). Universal PCR Master Mix No AmpErase UNG (ThermoFisher Scientific) was used for quantitative RT-PCR by the ABIPRISM 7900 HT Sequence Detection System (ThermoFisher Scientific). Quantification of mRNA expression was performed with a TaqMan® Gene Expression Assay (ThermoFisher Scientific) for each target gene (Table 1). RT-PCR was performed after 24 h incubation with ICs. Expression levels of the target genes (*tlr2*, *tlr3*, *tlr4*, *tlr7*, *tlr8*, *tlr9*, *interferon (ifn)-α*, *ifn-β*, *endothelin (et)-1, collagenIα1 (coIα1)* and *mmp-1*) were determined by the comparative Ct method normalizing the target to the endogenous gene (*gapdh*). Relative values of the target to the reference were expressed as the fold change (RQ). The optimal time point to evaluate the mRNA levels of colIα1 was set at 24 h based on a kinetics curve of the mRNA response to stimulation with TGF-β.

**Table 1:** TaqMan® Gene Expression Assays

Gene	TaqMan® Gene Expression ID
*tlr2*	Hs01872448_s1
*tlr3*	Hs01551078_m1
*tlr4*	Hs00152939_m1
*tlr7*	Hs01933259_s1
*tlr8*	Hs00152972_m1
*tlr9*	Hs00370913_s1
*ifn-α*	Hs00855471_g1
*ifn-β*	Hs01077958_s1
*et-1*	Hs00174961_m1
*colIα1*	Hs00164004_m1
*mmp-1*	Hs00899658_m1
*gapdh*	Hs99999905_m1

### Nuclear factor kappa B, p38 mitogen activated kinase and SAPK-JNK activation rate

Fibroblast monolayers were incubated with SSc-ICs, NHS-ICs, poly(I:C) and LPS. Total proteins were isolated using RIPA Lysis Buffer added to Protease and Phosphatase inhibitor cocktail (Sigma-Aldrich). Protein concentration was evaluated using the BCA Protein Assay Kit (ThermoFisher Scientific). Proteins were fractionated by NuPAGE BIS–TRIS by 4–12% SDS-polyacrylamide precast gel electrophoresis and transferred to nitrocellulose using iBlot Transfer Stacks Nitrocellulose (ThermoFisher Scientific). Membranes were blocked for 2 h at room temperature in PBS/0.05% Tween 20 (PT) (Bio-Rad Laboratories, Hercules, CA, USA) containing 5% nonfat milk powder (Mellin, Milan, Italy), and incubated with anti-human nuclear factor kappa B (NFκB), anti-human phosphorylated NFκB (pNFκB), anti-human p38 mitogen activated kinase (p38MAPK), anti-human phosphorylated p38MAPK (pp38MAPK), anti-human SAPK-JNK or anti-human phosphorylated SAPK-JNK (anti-pSAPK-JNK) antibodies (Cell Signaling Technology, Danvers, MA, USA). After washing, membranes were incubated in PT/5% nonfat milk powder plus HRP-conjugated secondary antibodies (MP Biomedicals, Santa Ana, CA, USA) and developed using the ECL Plus Detection System (ThermoFisher Scientific). Signals were detected using radiographic films (Kodak, Rochester, NY, USA). ImageJ software (LI-COR Biosciences, Lincoln, NE, USA) was used to analyze and quantify gels.

### SLE and APS immune complexes

The protein secretion of MMP-2 and Pro-CollagenIα1, the mRNA levels of *coIα1* and *mmp-1*, and the activation rate of NFκB, p38MAPK, p54SAPK-JNK and p46SAPK-JNK were also evaluated in response to stimulation with ICs from SLE and PAPS sera.

### DNase and RNase treatment

SSc-ICs were incubated for 1 h at 37 °C with recombinant DNase I or RNase (20 KU/ml and 8 μg/ml, respectively; Worthington Biochemical Corporation, Lakewood, NJ, USA) and then added to cells for 24 h. RT-PCR for *tlr2*, *tlr3*, *ifn*-*α* and *et-1* was then performed.

### NFκB and p38MAPK inhibitors

Cells were preincubated for 1 h at 37 °C with inhibitors of NFκB (MG-132, 20 μmol; Sigma-Aldrich) and p38MAPK (SB202190, 20 μmol; Cell Signaling Technology). The expression levels of IL-6 and the activation rates of NFκB and p38MAPK were assessed by western blot analysis. IL-8, TGF-β1 and Pro-CollagenIα1 were measured by commercial ELISA kits in culture supernatants.

### Scleroderma skin fibroblast cell culture

Dermal fibroblasts were isolated from two patients with dcSSc and cultured following the same procedures described for healthy fibroblasts. IL-6 and IL-8 secretion levels were assessed in culture supernatants after 48 h incubation with SSc-ICs, NHS-ICs or TLR synthetic agonists (LPS and poly(I:C)) by commercial ELISAs (R&D Systems).

### Statistical analysis

Descriptive statistics were used to calculate the mean and standard deviation (SD). Since our data were derived from in-vitro experiments conducted under high controlled conditions and originated from a high number of cells, ANOVA was used to compare different experimental conditions, and post-hoc comparisons were assessed by Dunnett’s test. With regards to nonhomogeneity of variance assumption, Welch’s correction was applied when required. Paired or unpaired *t* tests were performed to compare mean values between two groups. All analyses were performed with GraphPad Prism 5.01. *p* < 0.05 was considered significant.

The approval of the Institutional Review Board of Istituto G. Pini, Milan, Italy was obtained; all subjects provided written informed consent.

## Results

### Immune complex characterization

Quanta Lite C1q CIC ELISA confirmed that all PEG-precipitated preparations contained ICs. SSc preparations exhibited significantly higher IC amounts compared to NHS (50.77 ± 9.8 versus 5.95 ± 2.02, *p* < 0.01, *t* = 4.477; cutoff 10.8 Eq/ml).

**Fig. 1:**
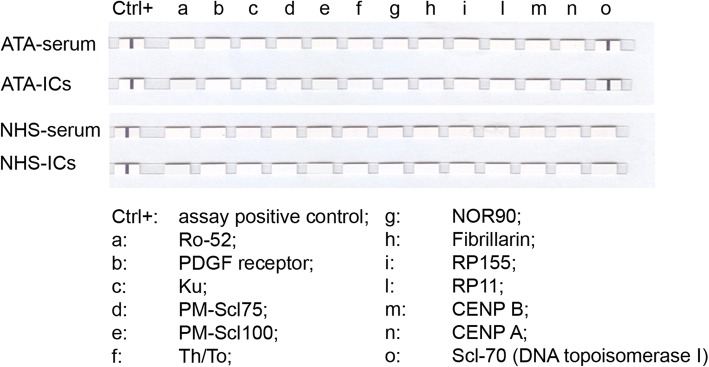
TaqMan® Gene Expression assays against SSc-specific antigens of PEG-precipitated ICs and corresponding sera evaluated by EUROLINE-SSc profile kit. One ATA-IC and one NHS-IC presented as representative assay. CTR+, assay-positive control. a, Ro-52; b, PDGF receptor; c, Ku; d, PM-Scl75; e, PM-Scl100; f, Th/To; g, NOR90; h, Fibrillarin; i, RP155; l, RP11; m, CENP B; n, CENP A; o, Scl-70 (DNA topoisomerase I). ATA anti-DNA topoisomerase I antibodies, IC immune complex, NHS normal healthy subjects

Using the EUROLINE-SSc profile kit on PEG-precipitated preparations, reactivity against SSc-specific antigens for each SSc-IC mirrored the corresponding serum autoantibody specificity. No reactivity was observed in NHS-ICs (Fig. 1).

**Fig. 2:**
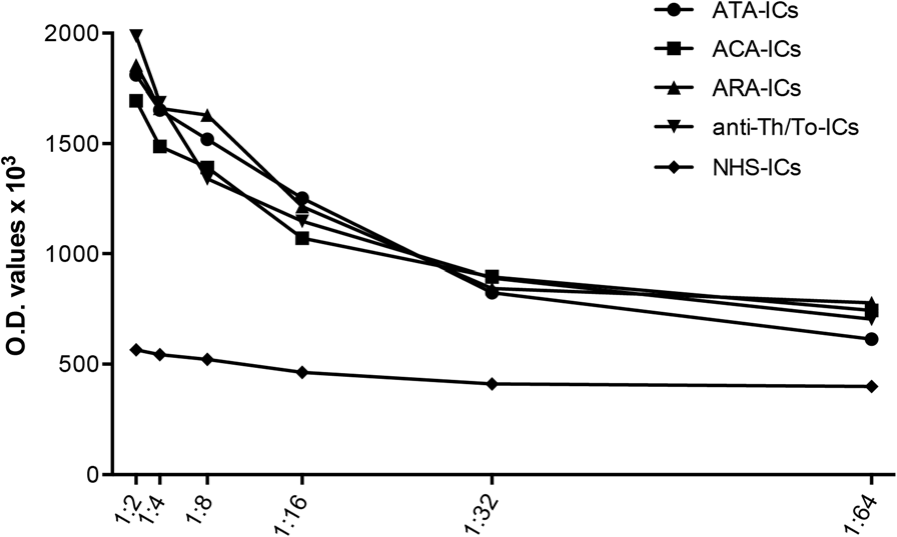
Dose–response dilution curve for ICAM-1 expression on fibroblast cell surface. Fibroblasts exposed to serial two-fold dilutions (from 1:2 to 1:64) of SSc-ICs and NHS-ICs, and ICAM-1 evaluated by cell ELISA. anti-Th/To anti-Th/To antibodies, ATA anti-DNA topoisomerase I antibodies, IC immune complex, NHS normal healthy subjects, OD optical density

To identify the optimal IC dilution in functional studies, a dose–response curve (1:2–1:64) was performed by cell ELISA for ICAM-1 expression on the fibroblast surface. A 1:2 dilution was selected as this allowed the highest response without affecting cell viability (Fig. 2).

### ICAM-1 expression

**Fig. 3:**
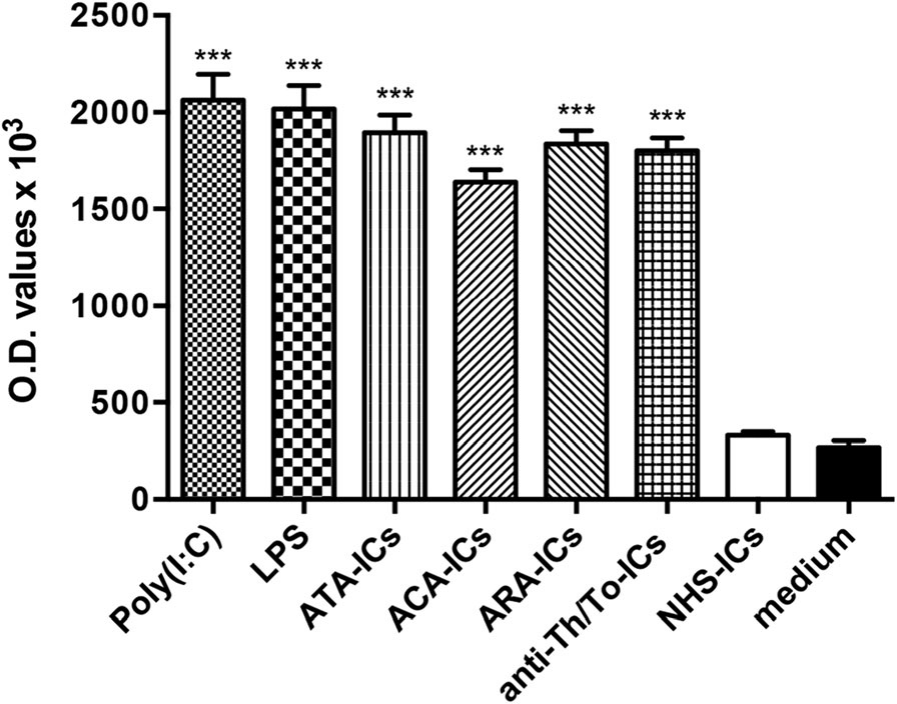
ICAM-1 expression on fibroblasts stimulated with SSc-ICs or NHS-ICs. Fibroblasts exposed to SSc-ICs or NHS-ICs (1:2 dilution). Poly(I:C) and LPS, at concentration of 1 μg/ml, used as positive controls. ***p < 0.0001 versus medium. ACA anti-centromeric protein antibodies, anti-Th/To anti-Th/To antibodies, ARA anti-RNA polymerase III antibodies, ATA anti-DNA topoisomerase I antibodies, IC immune complex, LPS lipopolysaccharide, NHS normal healthy subjects, OD optical density, poly(I:C) polyinosinic-polycytidylic acid

SSc-ICs significantly induced ICAM-1 expression on fibroblast monolayers compared to medium. No increase in ICAM-1 expression was observed with NHS-ICs. Poly(I:C) and LPS elicited a significant increase in ICAM-1 protein levels compared to medium (Fig. 3).

### IL-6, IL-8, MMP-2 and MCP-1 secretion

**Fig. 4:**
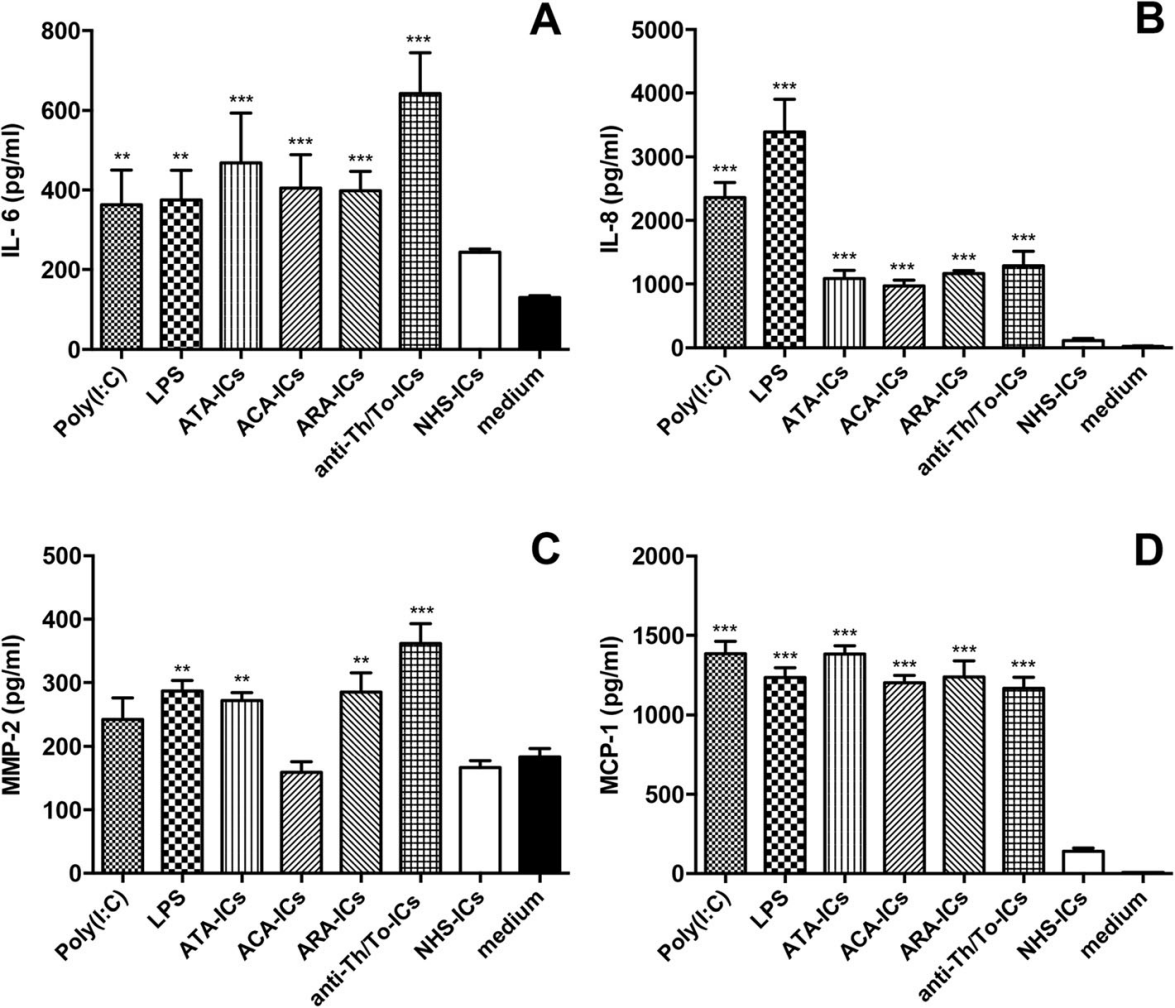
IL-6, IL-8, MMP-2 and MCP-1 levels in culture supernatants from fibroblasts incubated with SSc-ICs or NHS-ICs. Fibroblasts exposed to SSc-ICs or NHS-ICs (1:2 dilution). Poly(I:C) and LPS, at concentration of 1 μg/ml, used as positive controls. **a** IL-6; **b** IL-8; **c** MMP-2; **d** MCP-1. **p < 0.001, ***p < 0.0001 versus medium. ACA anti-centromeric protein antibodies, anti-Th/To anti-Th/To antibodies, ARA anti-RNA polymerase III antibodies, ATA anti-DNA topoisomerase I antibodies, IC immune complex, IL interleukin, LPS lipopolysaccharide, MCP monocyte chemoattractant protein, MMP matrix metalloproteinase, NHS normal healthy subjects, poly(I:C) polyinosinic-polycytidylic acid

All SSc-ICs, LPS and poly(I:C) upregulated IL-6 levels compared to medium. Conversely, NHS-ICs did not have any effect (Fig. 4a). All SSc-ICs, poly(I:C) and LPS elicited a significant rise in IL-8 levels compared to medium (Fig. 4b). Fibroblasts incubated with NHS-ICs exhibited IL-8 levels similar to cells treated with medium alone. ATA-ICs, ARA-ICs, anti-Th/To-ICs and LPS significantly upregulated MMP-2 levels compared to medium. ACA-ICs, poly(I:C) and NHS-ICs did not induce a significant increase in MMP-2 protein levels (Fig. 4c).

All SSc-ICs, poly(I:C) and LPS significantly upregulated MCP-1. NHS-ICs did not elicit a significant increase in MCP-1 protein levels (Fig. 4d).

### *et-1* and *ifn* mRNA expression

**Fig. 5:**
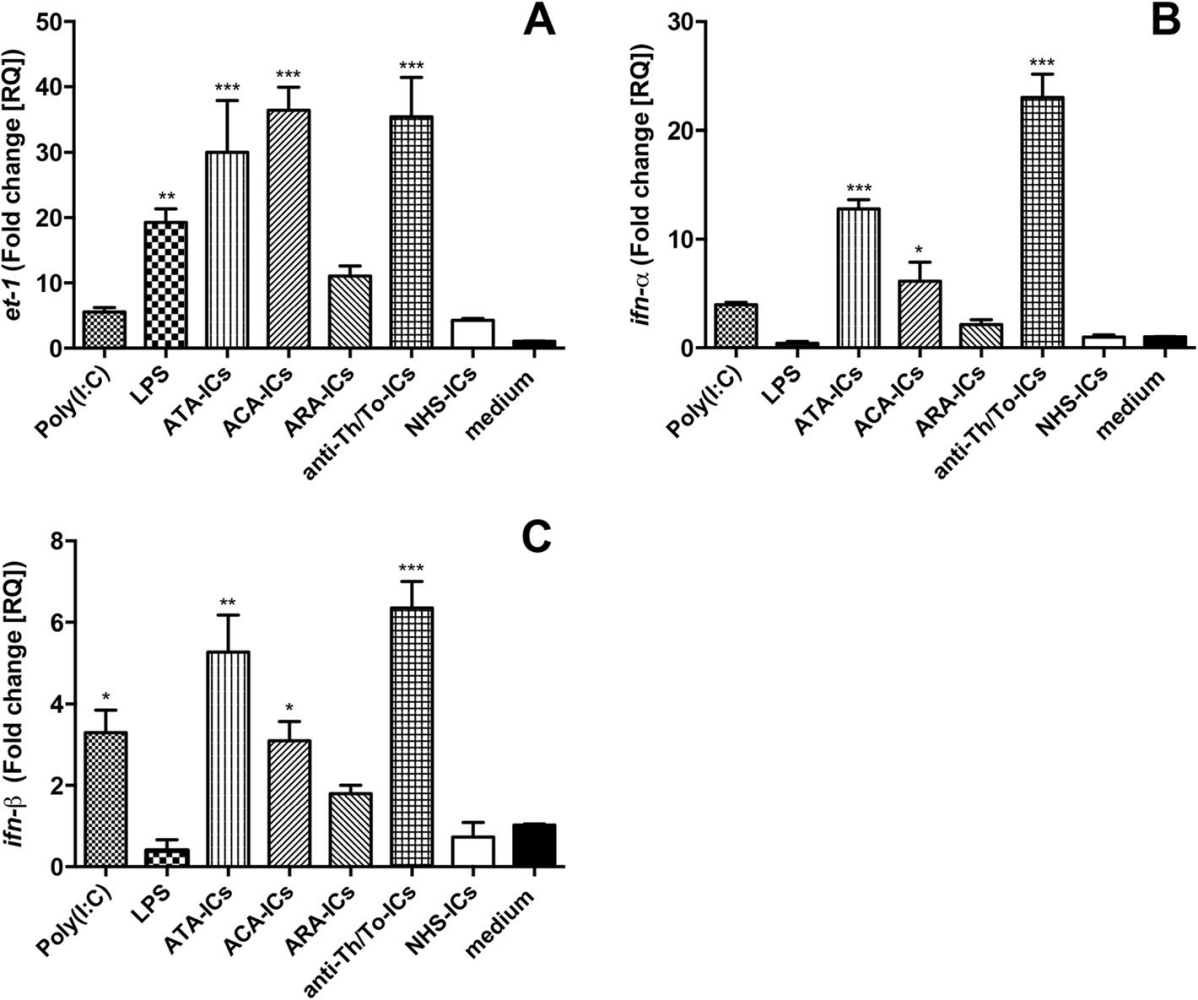
*et-1*, *ifn-α* and *ifn-β* mRNA expression levels in fibroblasts stimulated with SSc-ICs or NHS-ICs. Fibroblasts exposed to SSc-ICs or NHS-ICs (1:2 dilution). Poly(I:C) and LPS, at concentration of 1 μg/ml, used as controls. **a** *et-1*; **b** *ifn-α*; **c** *ifn-β*. *p < 0.01, **p < 0.001, ***p < 0.0001 versus medium. ACA anti-centromeric protein antibodies, anti-Th/To anti-Th/To antibodies, ARA anti-RNA polymerase III antibodies, ATA anti-DNA topoisomerase I antibodies, et-1 endothelin-1, IFN interferon, IC immune complex, LPS lipopolysaccharide, NHS normal healthy subjects, poly(I:C) polyinosinic-polycytidylic acid

LPS, ATA-ICs, ACA-ICs and anti-Th/To-ICs, but not ARA-ICs, significantly upregulated *et-1* levels compared to the medium, while NHS-ICs and poly(I:C) did not exert any effect (Fig. 5a).

ATA-ICs, ACA-ICs and anti-Th/To-ICs significantly upregulated mRNA levels of *ifn-α.* Poly(I:C), LPS, ARA-ICs and NHS-ICs did not significantly modulate *ifn-α* mRNA (Fig. 5b). Poly(I:C), ATA-ICs, ACA-ICs and anti-Th/To-ICs drove a significant increase of mRNA levels of *ifn-β.* Stimulation with LPS, ARA-ICs and NHS-ICs did not significantly affect mRNA of *ifn-β* compared to culture medium (Fig. 5c).

### TGF-β1 and Pro-CollagenIα1 secretion, and *colIα1* and *mmp-1* mRNA expression

**Fig. 6:**
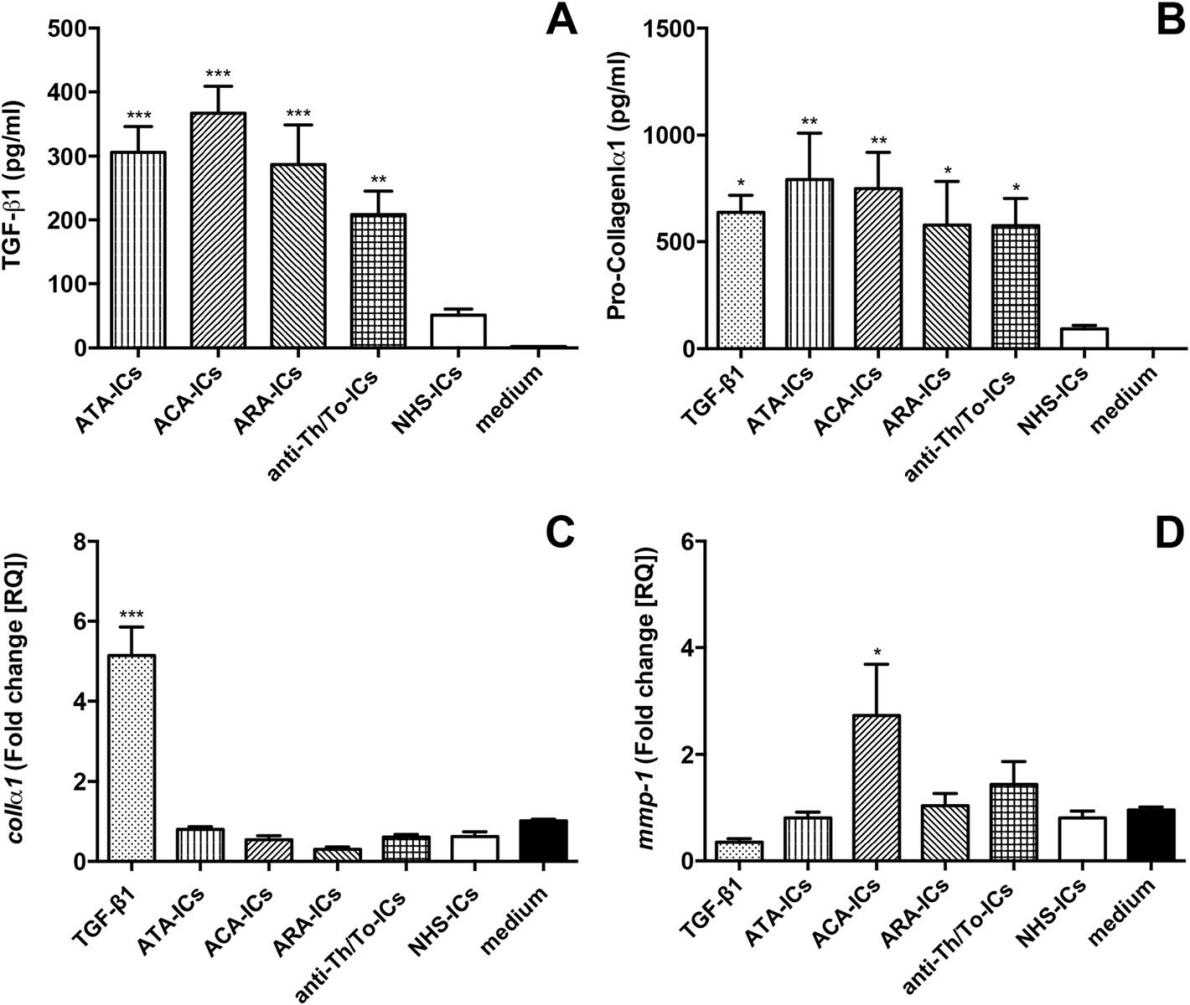
TGF-β1 and Pro-CollagenIα1 secretion and *colIα1* and *mmp-1* mRNA expression in fibroblasts stimulated with SSc-ICs or NHS-ICs. Fibroblasts exposed to SSc-ICs or NHS-ICs (1:2 dilution). TGF-β1 (10 ng/ml) used as positive control for collagen synthesis and secretion. **a** TGF-β1; **b** Pro-CollagenIα1; **c** *colIα1*; **d** *mmp-1*. *p < 0.01, **p < 0.001, ***p < 0.0001 versus medium. ACA anti-centromeric protein antibodies, anti-Th/To anti-Th/To antibodies, ARA anti-RNA polymerase III antibodies, ATA anti-DNA topoisomerase I antibodies, colIα1 collagenIα1, IC immune complex, MMP matrix metalloproteinase, NHS normal healthy subjects, TGF tumor growth factor

All SSc-ICs significantly increased TGF-β1 levels compared to medium alone, while NHS-ICs did not exert any effect (Fig. 6a). All SSc-ICs and TGF-β1 significantly upregulated Pro-CollagenIα1 secretion in supernatants compared to medium alone. NHS-ICs did not affect protein levels (Fig. 6b). SSc-ICs and NHS-ICs did not modulate *colIα1* mRNA expression. Conversely, TGF-β1 significantly increased *colIα1* mRNA levels (Fig. 6c). ACA-ICs drove a significant upregulation of *mmp-1* while all the other SSc-ICs as well as NHS-ICs and TGF-β1 did not significantly affect *mmp-1* mRNA levels (Fig. 6d).

### *tlr* mRNA expression

**Fig. 7:**
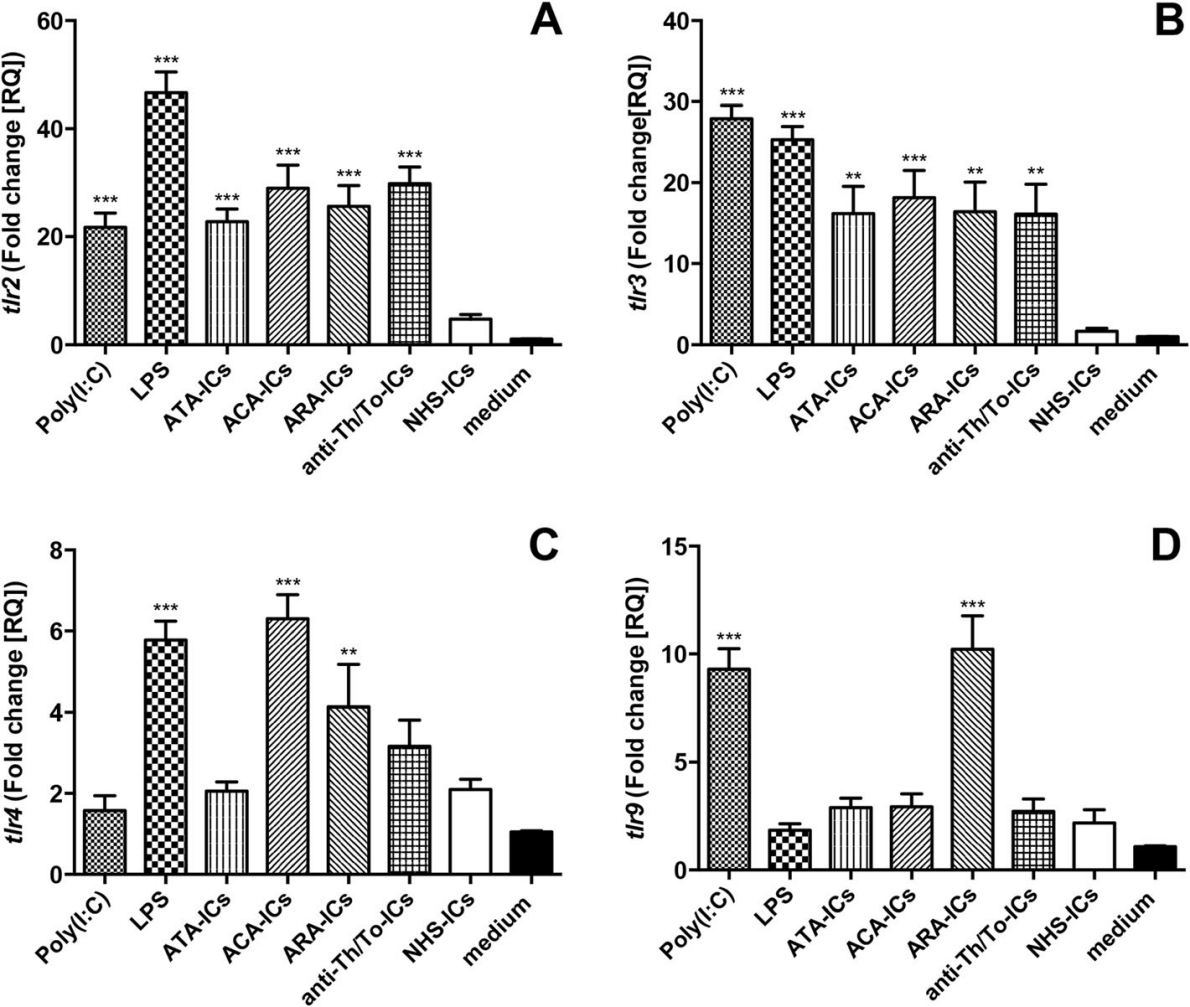
*tlr* mRNA expression levels in fibroblasts stimulated with SSc-ICs or control NHS-ICs. Fibroblasts exposed to SSc-ICs or NHS-ICs (1:2 dilution). Poly(I:C) and LPS, at concentration of 1 μg/ml, used as controls. **a** *tlr2*; **b** *tlr3*; **c** *tlr4*; **d** *tlr9*. **p < 0.001, ***p < 0.0001 versus medium. ACA anti-centromeric protein antibodies, anti-Th/To anti-Th/To antibodies, ARA anti-RNA polymerase III antibodies, ATA anti-DNA topoisomerase I antibodies, IC immune complex, LPS lipopolysaccharide, NHS normal healthy subjects, poly(I:C) polyinosinic-polycytidylic acid, TLR Toll-like receptor

All SSc-ICs, but not NHS-ICs, and both TLR agonists drove a significant increase in *tlr2* mRNA as compared to medium (Fig. 7a). Similarly, all SSc-ICs, but not NHS-ICs, induced a significant *tlr3* upregulation; an increase in *tlr3* mRNA was observed with poly(I:C) and LPS (Fig. 7b). LPS, ACA-ICs and ARA-ICs induced a significant upregulation in *tlr4* mRNA levels. Conversely, ATA-ICs, anti-Th/To-ICs, NHS-ICs and poly(I:C) did not affect *tlr4* mRNA levels (Fig. 7c). *tlr9* expression was significantly modulated by ARA-ICs and poly(I:C). Differently, ATA-ICs, ACA-ICs, anti-Th/To-ICs, NHS-ICs and LPS did not affect *tlr9* mRNA levels (Fig. 7d).

*tlr7* and *tlr8* mRNA could not be detected in fibroblasts.

### Intracellular signaling pathways

**Fig. 8:**
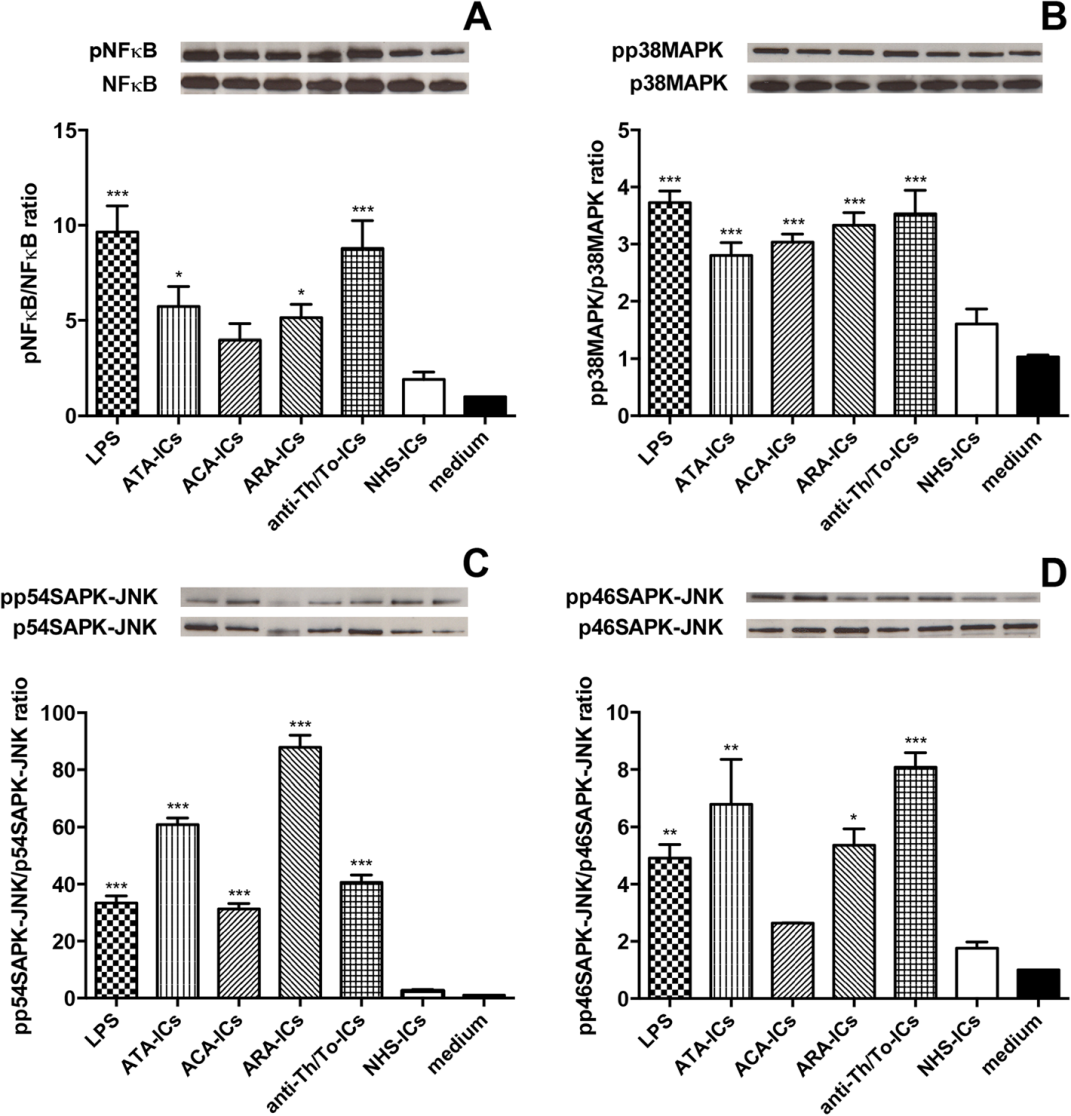
Intracellular signaling pathways in fibroblasts stimulated with SSc-ICs or NHS-ICs. Fibroblasts exposed to SSc-ICs or NHS-ICs (1:2 dilution). LPS (1 μg/ml) used as control. **a** pNFκB/NFκB; **b** pp38MAPK/p38MAPK; **c** pp54SAPK-JNK/p54SAPK-JNK; **d** pp46SAPK-JNK/p46SAPK-JNK. Results expressed as ratio of phosphorylated to nonphosphorylated forms, evaluated using ImageJ software. Western blot images representative of single experiment. *p < 0.01, **p < 0.001, ***p < 0.0001 versus medium. ACA anti-centromeric protein antibodies, anti-Th/To anti-Th/To antibodies, ARA anti-RNA polymerase III antibodies, ATA anti-DNA topoisomerase I antibodies, IC immune complex, LPS lipopolysaccharide, MAPK mitogen activated kinase, NHS normal healthy subjects, NFκB nuclear factor kappa B, pNFκB phosphorylated NFκB, pp38MAPK phosphorylated p38MAPK, pp54SAPK-JNK phosphorylated p54SAPK-JNK, pp46SAPK-JNK phosphorylated p46SAPK-JNK

ATA-ICs, ARA-ICs, anti-Th/To-ICs and LPS significantly activated NFκB compared to the medium. Conversely, ACA-ICs and NHS-ICs did not elicit NFκB phosphorylation (Fig. 8a). All SSc-ICs, but not NHS-ICs, and LPS activated p38MAPK and p54SAPK-JNK (Fig. 8b, c). ATA-ICs, ARA-ICs, anti-Th/To-ICs and LPS induced a significant increased phosphorylation rate of p46SAPK-JNK. ACA-ICs and NHS-ICs did not exert any effect on the phosphorylation rate of p46SAPK-JNK (Fig. 8d).

### Immune complexes from SLE and PAPS

**Fig. 9:**
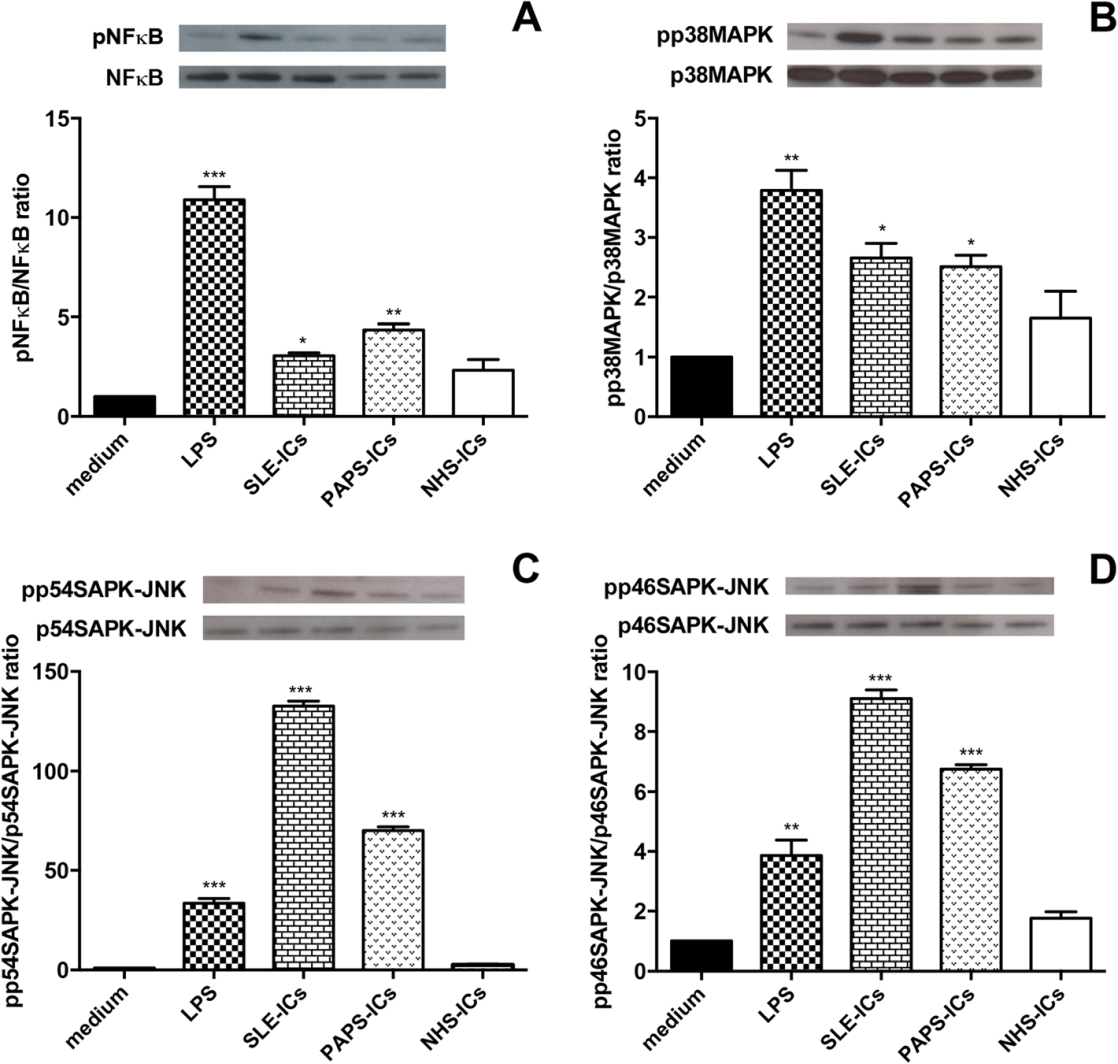
Intracellular signaling pathways in fibroblasts stimulated with SLE-ICs, PAPS-ICs or NHS-ICs. Fibroblasts exposed to SLE-ICs, PAPS-ICs or NHS-ICs (1:2 dilution). LPS (1 μg/ml) used as control. **a** pNFκB/NFκB; **b** pp38MAPK/p38MAPK; **c** pp54SAPK-JNK/p54SAPK-JNK; **d** pp46SAPK-JNK/p46SAPK-JNK. Results expressed as ratio of phosphorylated to nonphosphorylated forms, evaluated using ImageJ software. Western blot images representative of single experiment. *p < 0.01, **p < 0.001, ***p < 0.0001 versus medium. IC immune complex, LPS lipopolysaccharide, MAPK mitogen activated kinase, NHS normal healthy subjects, NFκB nuclear factor kappa B, pNFκB phosphorylated NFκB, pp38MAPK phosphorylated p38MAPK, pp54SAPK-JNK phosphorylated p54SAPK-JNK, pp46SAPK-JNK phosphorylated p46SAPK-JNK, PAPS primary anti-phospholipid syndrome, SLE systemic lupus erythematosus

**Fig. 10:**
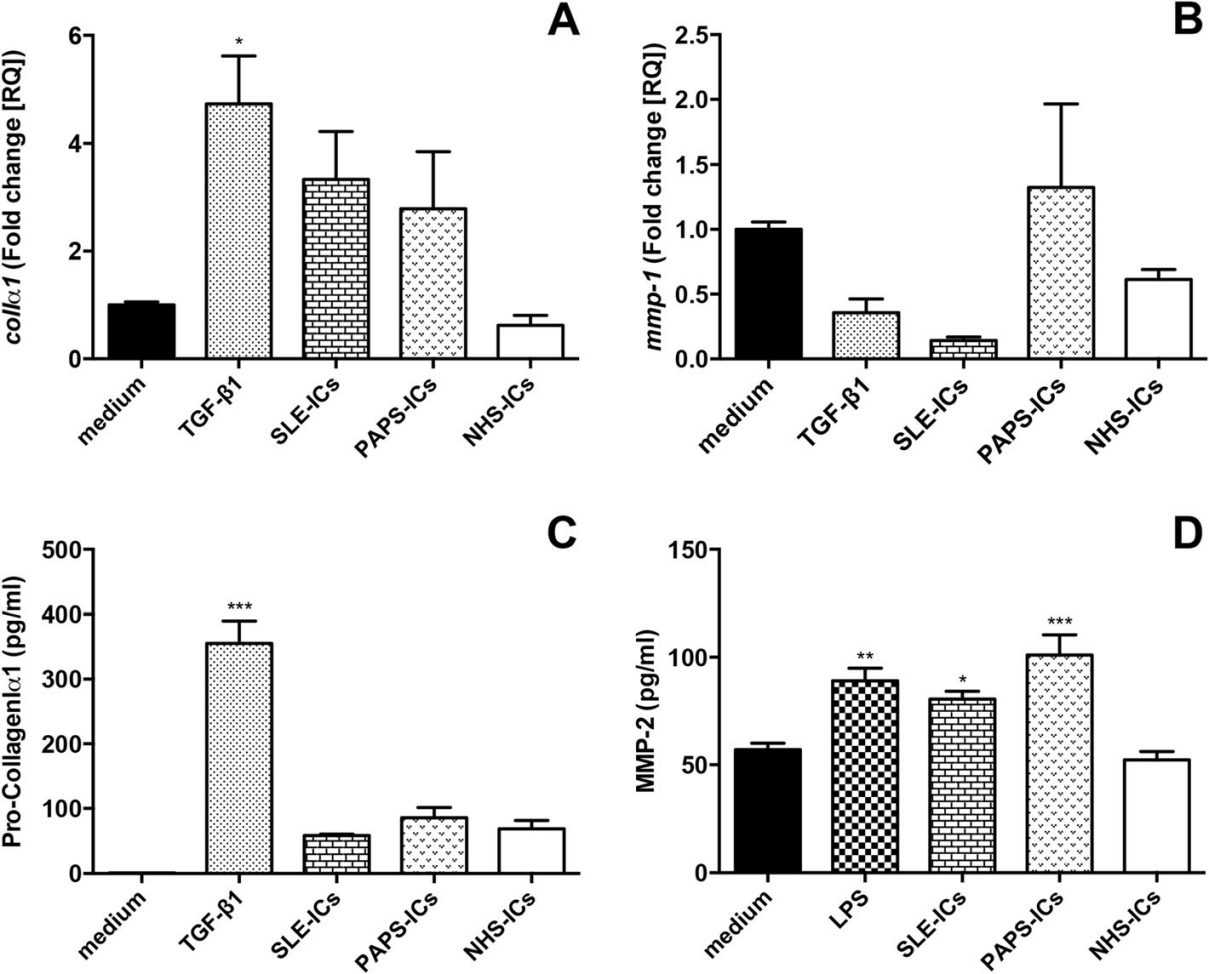
*colIα1* and *mmp-1* mRNA expression and Pro-CollagenIα1 and MMP-2 secretion in fibroblasts stimulated with SLE-ICs, PAPS-ICs or NHS-ICs. Fibroblasts exposed to PAPS-ICs, SLE-ICs or NHS-ICs (1:2 dilution). TGF-β1 (10 ng/ml) and LPS (1 μg/ml) used as positive control for collagen synthesis and secretion. **a** *colIα1*; **b** *mmp-1*; **c** Pro-CollagenIα1; **d** MMP-2. *p < 0.01, **p < 0.001, ***p < 0.0001 versus medium. colIα1 collagenIα1, IC immune complex, LPS lipopolysaccharide, MMP matrix metalloproteinase, NHS normal healthy subjects, PAPS primary anti-phospholipid syndrome, SLE systemic lupus erythematosus, TGF tumor growth factor

SLE-ICs and PAPS-ICs phosphorylated NFκB (Fig. 9a), p38MAPK (Fig. 9b), p54SAPK-JNK (Fig. 9c) and p46SAPK-JNK (Fig. 9d). SLE-ICs and PAPS-ICs did not induce a significant upregulation of the mRNA levels of c*olIα1* (Fig. 10a) and *mmp-1* (Fig. 10b) or of the secretion of Pro-CollagenIα1 (Fig. 10c). SLE-ICs and PAPS-ICs, as well as LPS, significantly upregulated MMP-2 secretion compared to medium. NHS-ICs did not significantly affect MMP-2 levels (Fig. 10d).

### Nucleic acid content

SSc-ICs contained a higher amount of nucleic acids compared to NHS-ICs (ssDNA 55.75 ± 16.41 ng/μl versus 43.11 ± 12.36 ng/μl (*p* = 0.0470, *t* = 2.075); dsDNA 81.06 ± 29.84 ng/μl versus 49.13 ± 13.51 ng/μl (*p* = 0.0087, *t* = 2.867); RNA 62.70 ± 22.67 ng/μl versus 37.14 ± 10.42 ng/μl (*p* = 0.0086, *t* = 2.852)).

**Fig. 11:**
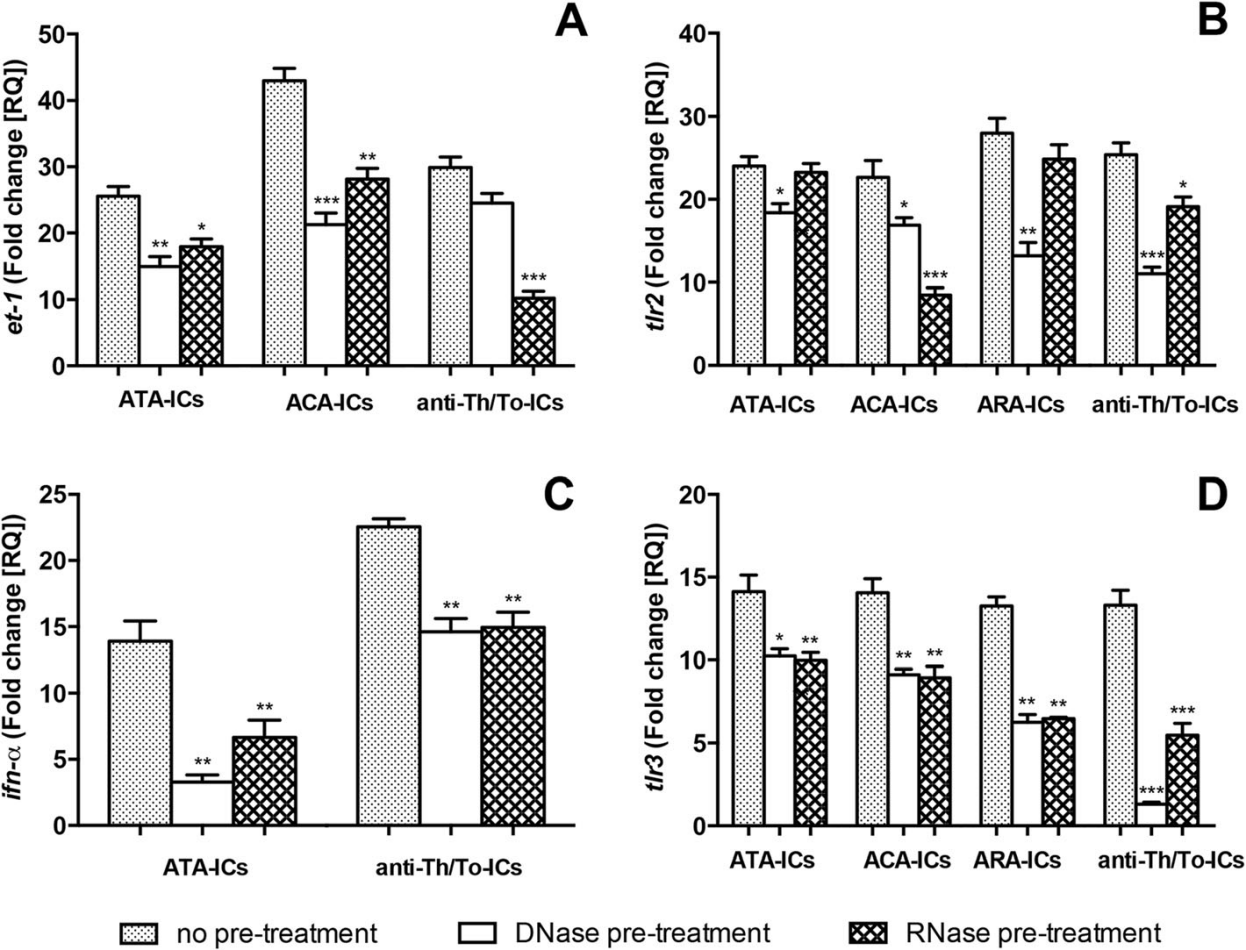
*et-1*, *tlr2* and *tlr3* expression levels in fibroblasts stimulated with SSc-ICs or NHS-ICs pretreated with DNase/RNase. SSc-ICs treated with DNase I (20 KU/ml) or RNase (8 μg/ml) and then added to fibroblast cultures. **a** ATA-ICs, ACA-ICs and anti-Th/To-ICs on *et-1*; **b** ATA-ICs, ACA-ICs, ARA-ICs and anti-Th/To-ICs on *tlr2*; **c** ATA-ICs and anti-Th/To-ICs on *ifn-α*; **d** ATA-ICs, ACA-ICs, ARA-ICs and anti-Th/To-ICs on *tlr3*. *p < 0.01, **p < 0.001, ***p < 0.0001 versus medium. ACA anti-centromeric protein antibodies, anti-Th/To anti-Th/To antibodies, ARA anti-RNA polymerase III antibodies, ATA anti-DNA topoisomerase I antibodies, et-1 endothelin-1, IC immune complex, IFN interferon, TLR Toll-like receptor

DNase I and RNase pretreatment modulated the expression of study mediators induced by SSc-ICs. Both DNase I and RNase treatments prevented mRNA upregulation of *et-1* by ATA-ICs and ACA-ICs (Fig. 11a), *tlr2* by ACA-ICs and anti-Th/To-ICs (Fig. 11b), *ifn*-α by ATA-ICs and anti-Th/To-ICs (Fig. 11c), and *tlr3* by ATA-ICs, ACA-ICs, ARA-ICs and anti-Th/To-ICs (Fig. 11d). *et-1* upregulation by anti-Th/To-ICs was modulated by RNase only (Fig. 11a); *tlr2* mRNA expression enhancement observed with ATA-ICs and ARA-ICs was reduced by DNase I only (Fig. 11b). DNase I and RNase treatments did not significantly affect study mediators when cells were incubated with NHS-ICs.

### NFκB and p38MAPK inhibitors

**Fig. 12:**
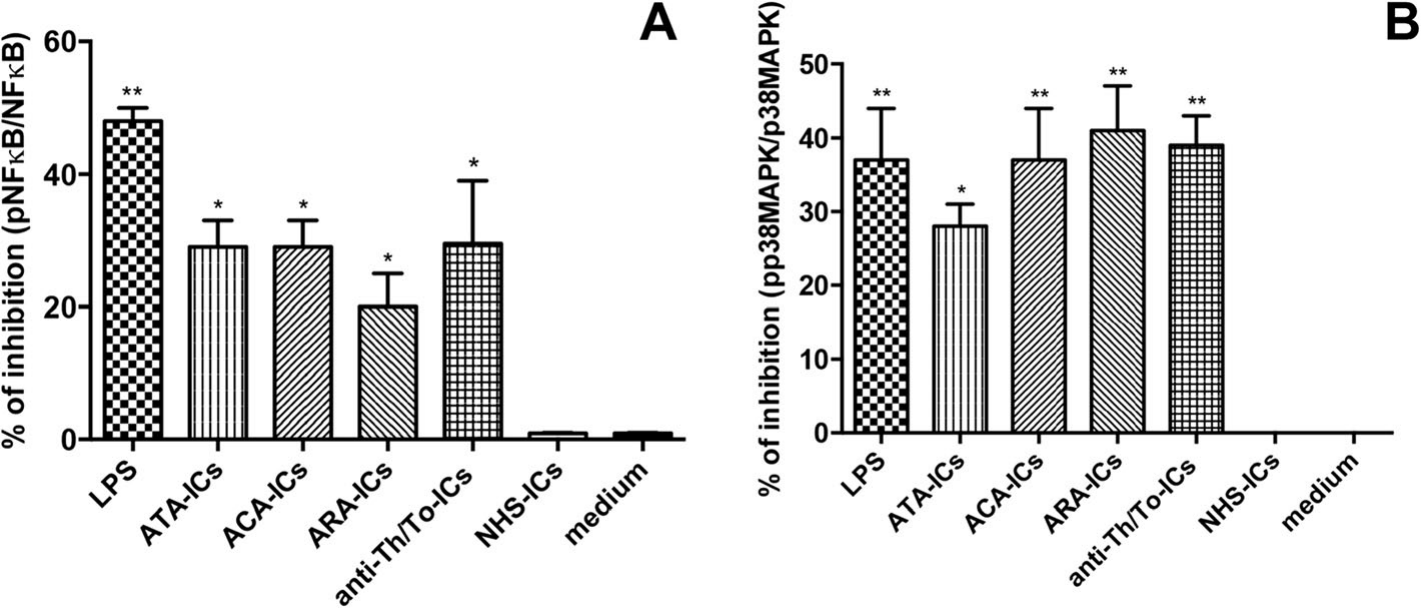
Confirmation of efficacy of NFκB and p38MAPK inhibitors by western blot analysis. Cells preincubated for 1 h at 37 °C with inhibitors of NFκB and p38MAPK. Fibroblasts exposed to SSc-ICs or NHS-ICs (1:2 dilution). LPS (1 μg/ml) used as control. Results expressed as percentage of inhibition of activated (**a**) NFκB and (**b**) p38MAPK (expressed as ratio of phosphorylated to nonphosphorylated forms). *p < 0.01, **p < 0.001 versus medium. ACA anti-centromeric protein antibodies, anti-Th/To anti-Th/To antibodies, ARA anti-RNA polymerase III antibodies, ATA anti-DNA topoisomerase I antibodies, IC immune complex, LPS lipopolysaccharide, NFκB nuclear factor kappa B, NHS normal healthy subjects, MAPK mitogen activated kinase, pp38MAPK phosphorylated p38MAPK

The efficacy of NFκB and p38MAPK inhibitors was confirmed by western blot analysis (Fig. 12a, b). When cells were treated with NFκB inhibitor, the expression levels of IL-6 in response to stimulation with LPS as well as all SSc-ICs and NHS-ICs were not affected.

**Fig. 13:**
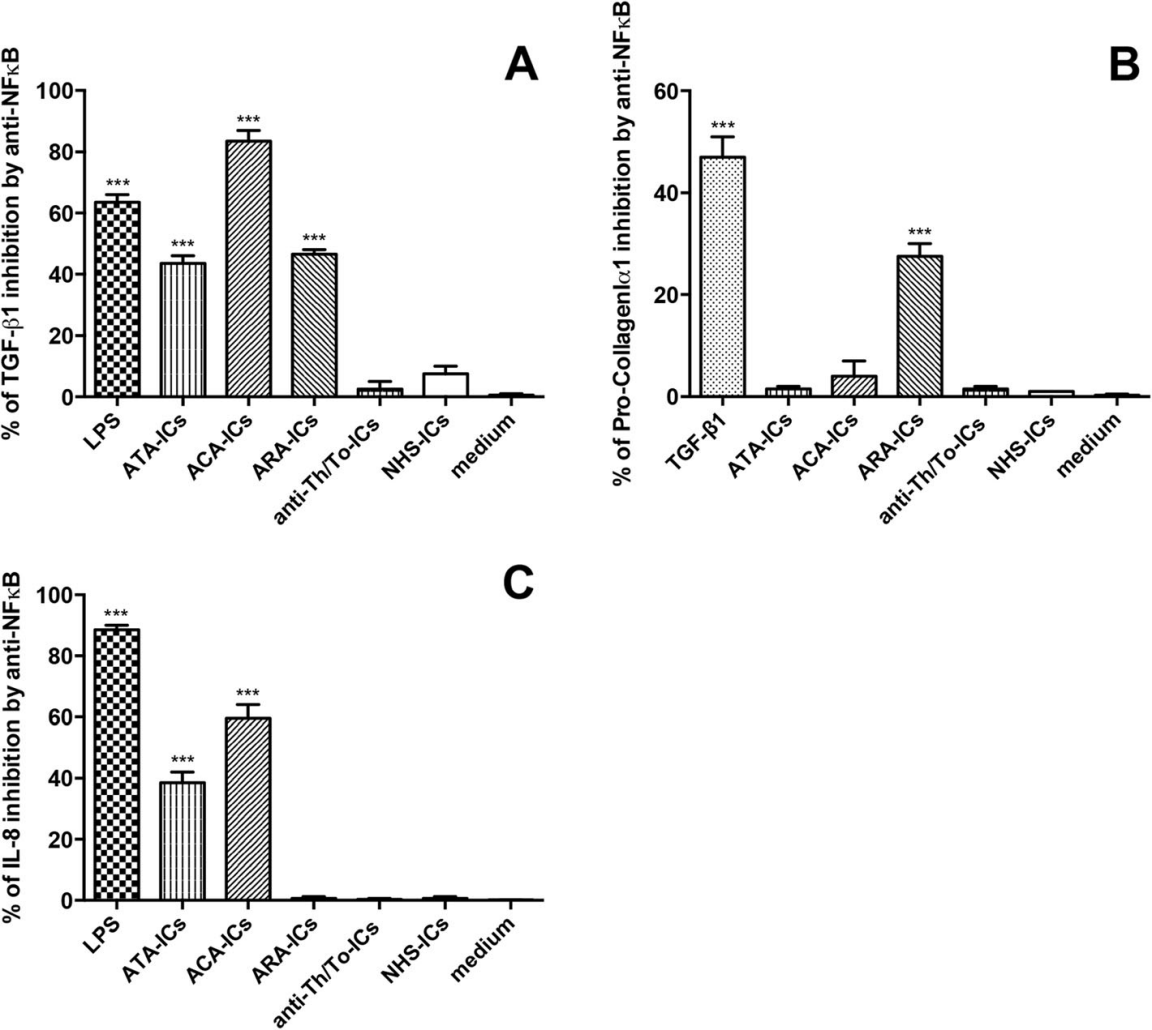
TGF-β1, Pro-collagenIα1 and IL-8 in fibroblasts pretreated with NFκB inhibitor and incubated with SSc-ICs or NHS-ICs. Fibroblasts pretreated with MG-132 (20 μmol), an NFκB inhibitor, and then exposed to SSc-ICs or NHS-ICs (1:2 dilution). LPS (1 μg/ml) and TGF-β1 (10 ng/ml) used as positive controls. Results expressed as percentage of inhibition of **a** TGF-β1, **b** Pro-CollagenIα1 and **c** IL-8 in untreated versus MG-132-treated cells. ***p < 0.0001 versus medium. ACA anti-centromeric protein antibodies, anti-Th/To anti-Th/To antibodies, ARA anti-RNA polymerase III antibodies, ATA anti-DNA topoisomerase I antibodies, IC immune complex, IL interleukin, LPS lipopolysaccharide, NFκB nuclear factor kappa B, NHS normal healthy subjects, TGF tumor growth factor

The inhibition of NFκB resulted in the significant modulation of TGF-β1 in response to LPS, ATA-ICs, ACA-ICs and ARA-ICs but not anti-Th/To-ICs and NHS-ICs (Fig. 13a). Pretreatment with NFκB inhibitor significantly downregulated the expression levels of Pro-CollagenIα1 induced by TGF-β1 and ARA-ICs but not ATA-ICs, ACA-ICs, anti-Th/To-ICs and NHS-ICs (Fig. 13b). Pretreatment with NFκB inhibitor led to the significant downregulation of the secretion levels of IL-8 induced by LPS, ATA-ICs and ACA-ICs but not ARA-ICs, anti-Th/To-ICs and NHS-ICs (Fig. 13c).

**Fig. 14:**
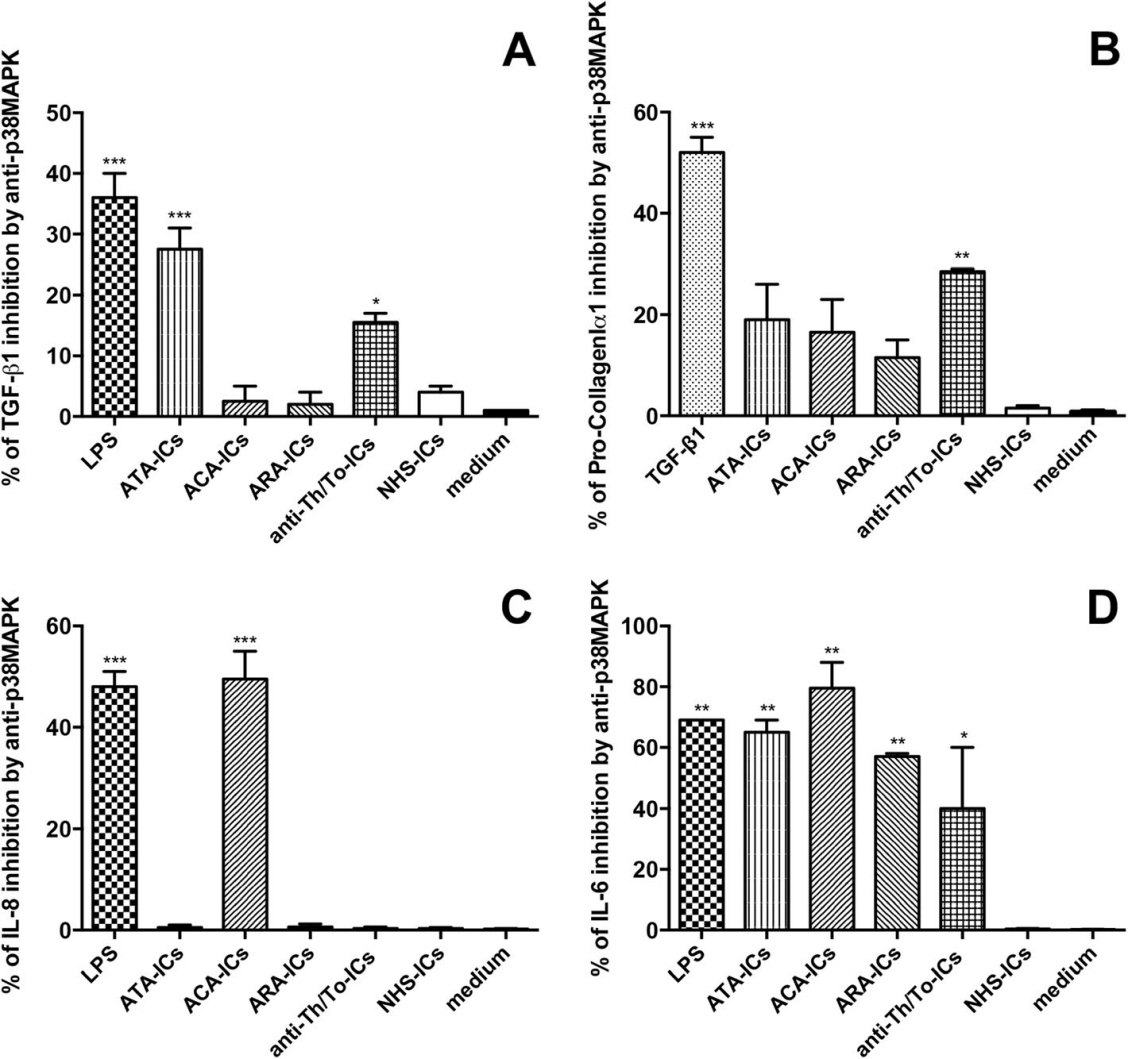
TGF-β1, Pro-collagenIα1, IL-8 and IL-6 in fibroblasts pretreated with p38MAPK inhibitor and incubated with SSc-ICs or NHS-ICs. Fibroblasts pretreated with SB202190 (20 μmol), a p38MAPK inhibitor, and then exposed to SSc-ICs or NHS-ICs (1:2 dilution). LPS (1 μg/ml) and TGF-β1 (10 ng/ml) used as positive controls. Results expressed as percentage of inhibition of **a** TGF-β1, **b** Pro-CollagenIα1, **c** IL-8 and **d** IL-6 in untreated versus SB202190-treated cells. *p < 0.01, **p < 0.001, ***p < 0.0001 versus medium. ACA anti-centromeric protein antibodies, anti-Th/To anti-Th/To antibodies, ARA anti-RNA polymerase III antibodies, ATA anti-DNA topoisomerase I antibodies, IC immune complex, IL interleukin, LPS lipopolysaccharide, NHS normal healthy subjects, MAPK p38 mitogen activated kinase, TGF tumor growth factor

The inhibition of p38MAPK led to the significant modulation of TGF-β1 in response to LPS, ATA-ICs and anti-Th/To-ICs but not ACA-ICs, ARA-ICs and NHS-ICs (Fig. 14a). Pretreatment with p38MAPK inhibitor significantly downregulated the expression levels of Pro-CollagenIα1 induced by TGF-β1 and anti-Th/To-ICs, but not ATA-ICs, ACA-ICs, ARA-ICs and NHS-ICs (Fig. 14b). Pretreatment with p38MAPK inhibitor resulted in the significant downregulation of IL-8 induced by LPS and ACA-ICs but not ATA-ICs, ARA-ICs, anti-Th/To-ICs and NHS-ICs (Fig. 14c). When cells were pretreated with p38MAPK inhibitor, the expression of IL-6 induced by LPS, ATA-ICs, ACA-ICs, ARA-ICs and anti-Th/To-ICs, but not NHS-ICs, was significantly affected (Fig. 14d).

### IL-6 and IL-8 secretion in scleroderma fibroblasts

**Fig. 15:**
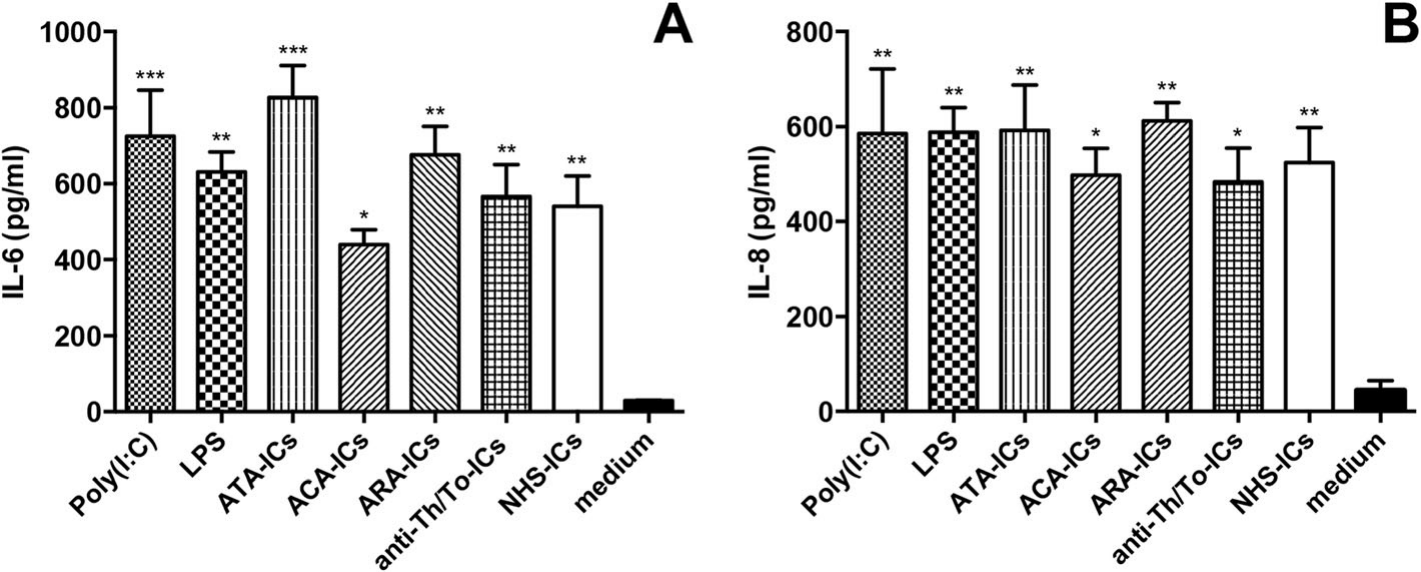
IL-6 and IL-8 in culture supernatants from dcSSc fibroblasts incubated with SSc-ICs or NHS-ICs. dcSSc fibroblasts exposed to SSc-ICs or NHS-ICs (1:2 dilution). Poly(I:C) and LPS, at concentration of 1 μg/ml, used as positive controls. **a** IL-6; **b** IL-8. *p < 0.01, **p < 0.001, ***p < 0.0001 versus medium. ACA anti-centromeric protein antibodies, anti-Th/To anti-Th/To antibodies, ARA anti-RNA polymerase III antibodies, ATA anti-DNA topoisomerase I antibodies, IC immune complex, IL interleukin, LPS lipopolysaccharide, NHS normal healthy subjects, poly(I:C) polyinosinic-polycytidylic acid

In fibroblasts from subjects with dcSSc, all ICs—those from SSc patients as well as NHS—upregulated both IL-6 and IL-8 as compared to medium. Even poly(I:C) and LPS elicited a significant raise in IL-6 and IL-8 levels compared to medium (Fig. 15a, b, respectively).

## Discussion

To our knowledge, this is the first study investigating in vitro the pathogenic potential of ICs containing SSc-specific antibodies. The first relevant observation that emerged in this work consists of the confirmation of IC content in all PEG-precipitated preparations, with significantly higher IC amounts in the samples from SSc patients compared to NHS. Consistently, early works evaluating circulating ICs in serum samples from SSc patients evinced a positivity rate similar to SLE [24–27]. To note, the antigenic reactivity of each PEG-precipitated preparation mirrored the autoantibody specificity of the original serum, thus confirming that ICs contain scleroderma-specific autoantibodies. Most importantly, functional experiments showed that SSc-ICs can affect the functionality of skin fibroblasts, the main effectors of tissue fibrosis. In particular, the incubation with SSc-ICs modulated several molecules involved in the three cardinal scleroderma pathophysiologic processes: vascular dysfunction (ET-1 and IL-8), inflammation (ICAM-1, IL-6, IFNs and MCP-1) and fibrosis (TGF-β1 and Pro-CollagenIα1). Noteworthy, SSc-ICs affected the protein but not the mRNA levels of collagen, possibly due to the posttranscriptional regulatory mechanism of collagen metabolism [28]; this hypothesis should be confirmed in future experiments. Another aspect that still needs elucidation is the role of type I IFNs. In our study, all SSc-ICs except ARA-ICs drove an interferogenic response, consistent with previous observations, such as the high IFN levels in scleroderma sera and skin samples and the IFN signature in peripheral blood cells and tissue macrophages from SSc patients [29, 30]. In particular, ATA-ICs significantly increased both IFN-α and IFN-β levels, in agreement with the already reported upregulation of IFNs elicited by ATA-positive sera in peripheral blood mononuclear cells [31, 32]. If the earlier cited authors proposed IFNs as early and prominent profibrotic mediators, it should be remembered that other investigators claimed an antifibrotic effect for IFNs, warranting further investigations [33, 34].

Our experiments suggest that SSc-ICs exert a specific pathogenic role in scleroderma, as compared to disease control ICs. Indeed, ICs from patients with autoimmune conditions other than SSc were tested: PAPS and SLE were identified as prototypical diseases. SLE is a systemic autoimmune disease characterized by a flourishing autoantibody production and a polymorphic clinical presentation [35]. APS is a systemic autoimmune condition characterized by vascular thrombosis and/or obstetric complications, in the persistent presence of circulating anti-phospholipid antibodies [36]. In both diseases, autoantibodies have been shown to exert a pathogenic role as well as a diagnostic one [37, 38]. Interestingly, in our study, SLE-ICs and PAPS-ICs did not modulate molecules directly involved in fibrogenesis such as *mmp-1*, *colIα1* and Pro-CollagenIα1. Both PAPS-ICs and SLE-ICs elicited a significant activation of intracellular mediators which are known to be involved in PAPS and SLE pathogenesis, such as NFκB, p38MAPK and SAPK-JNK [37, 38].

According to our data, the pathogenic effects of SSc-ICs on fibroblasts might be mediated by innate immunity sensors as TLRs. This hypothesis would allow overcoming one of the strongest objections against the pathogenicity of SSc autoantibodies, the intracellular localization of target antigens. Indeed, while dendritic and B cells could engage SSc autoantibodies via Fcγ receptor (FcγR), this is not the case of skin fibroblasts, which lack FcγR [39]. To note, genetic, in-vitro, in-vivo and ex-vivo findings are increasingly acknowledging TLRs as master players in SSc pathogenesis [40]. Consistently, SSc-ICs upregulated TLR expression, although to a lower extent for *tlr9*. The recruitment of intracellular mediators downstream of TLRs was observed for SSc-ICs but not NHS-ICs, further suggesting the potential involvement of TLRs in driving the SSc-IC signal. We further investigated the contribution of intracellular mediators by pretreating fibroblast cells with NFκB and p38MAPK inhibitors. Data suggested that SSc-ICs might engage intracellular signaling pathways differently: response to anti-Th/To-ICs was preferentially mediated by p38MAPK. Conversely, stimulation with ATA-ICs, ACA-ICs and ARA-ICs appears to engage both intracellular mediators.

It could thus be proposed that TLRs on target cells might interact with SSc-ICs via the nucleic acid fragments. TLR2, which does not recognize nucleic acids, might recognize HMGB-1, which is incorporated in ICs and acts as an agonist for both TLR2 and TLR4 [41, 42]. Our working hypothesis is suggested by the significant enrichment in nucleic acids of SSc-ICs compared to NHS-ICs. As further support, DNase I and RNase pretreatment prevented the upregulation of mediators induced by SSc-ICs, consistent with previous observations [31]. These data fit well with the recent evidence of elevated serum nucleosomes (histone proteins wrapped with DNA fragments) among SSc patients [43]. Nucleic acids embedded in ICs might be of endogenous nature: DNA and RNA residues are released from damaged and necrotic self-cells. Noteworthy, SSc patients exhibit increased DNA damage in peripheral blood cells [44]; the gene coding for *DNASE1L3*, an enzyme involved in DNA fragmentation during apoptosis, is one of the strongest susceptibility loci for SSc [45]. Nucleic acids from pathogens might also be included in ICs; interestingly, Epstein–Barr virus (EBV) was shown to infect most fibroblasts and endothelial cells in the skin of SSc patients [46].

Noteworthy, when fibroblasts from patients with dcSSc were used as an in-vitro model, we observed a significant difference in the modulation of IL-6 and IL-8 secretion levels in response to stimulation with all SSc-ICs as well as NHS-ICs. These findings are in agreement with the well-known hyperresponsiveness of scleroderma fibroblasts even to aspecific stimuli, prompting us to focus our research on healthy skin fibroblasts in order to reproduce the initiator phase of the disease.

As a whole, our findings allowed us to formulate a comprehensive hypothesis which postulates scleroderma-specific autoantibodies embedded in SSc-ICs as novel players in disease pathogenesis. The proposed pathogenic relevance of SSc-ICs fits well with the evidence that autoantibody positivity is the strongest predictor of progression into full-blown SSc [5]. Further support for the pathogenicity of autoantibodies comes from the striking temporal clustering and casual link between solid tumors and ARA-positive SSc [47, 48]. Indeed, the POLR3A locus, coding for the antigenic target of ARA, was altered in cancer tissue specimens from SSc patients carrying ARA, leading to the synthesis of an immunogenic enzyme resulting in T-cell-driven ARA production and SSc onset [49].

We reckon that several functional autoantibodies have already been described in SSc: anti-fibroblast antibodies (AFA), anti-endothelial cell antibodies, antibodies against platelet-derived growth factor receptor, etc. However, differently from SSc-specific antibodies, most of these functional autoantibodies can be detected in a minority of patients’ sera and present a poor specificity for scleroderma, being positive in many other autoimmune diseases and even in NHS [50]. In particular, AFA have been reported to upregulate ICAM-1, IL-6, IL-1α, IL-1β, CCL2, CXCL8 and MMP-1, with partial exploitation of TLR4, whereas collagen and tissue inhibitor of MMP-1 were not affected [51, 52]. Interestingly, it has been suggested that anti-fibroblast activity might be mediated by ATA: AFA purified from SSc patients strongly reacted with topoisomerase I and AFA positivity at high titers correlated with pulmonary involvement and death [7].

We acknowledge that our work presents intrinsic limitations. Being an in-vitro study, it might be oversimplistic, not allowing adequate reproduction of the complexity of scleroderma pathogenesis. It would be intriguing to test the effects of scleroderma ICs on endothelial cells, macrophages and lymphocytes, some of the mosaic of cells contributing to the many processes that underpin fibrogenesis. In this regard, we have preliminarily observed that endothelial cells stimulated with SSc-ICs secrete a significantly higher amount of TGF-β1, which might in turn act on fibroblasts, hence promoting the acquisition of a profibrotic phenotype (unpublished data). Indeed, further in-vitro experiments using cell cocultures (e.g., endothelial cells and fibroblasts, fibroblasts and macrophages, etc.) should be performed in order to reproduce the multifaceted cellular orchestra implicated in scleroderma etiopathogenesis. We reckon that a major limitation of this study is the scarce number of samples for each autoantibody specificity [53]. However, the limited patient cohort does not impinge the robustness of the conclusions of this study, whose aim was to provide the first proof-of-concept of IC involvement in the pathophysiology of SSc. In addition, some of the autoantibodies under investigation, such as ARA and anti-Th/To, are quite uncommon in the Italian SSc population [53], thus preventing the collection of a broader number of samples. Nevertheless, we believe this study offers important insights into scleroderma pathophysiology: the effects of SSc-ICs should be appropriately confirmed using samples from a wider cohort of SSc patients and the intracellular mediators engaged by SSc-ICs should be further characterized.

It would be tempting to postulate that the different modulation of some study mediators that emerged upon stimulation with the different scleroderma ICs might account for the characteristic clinical phenotype associated with each autoantibody profile; however, the differential response to SSc-ICs reacting with various antigens might be ascribed to the IC contents of the preparations. Unfortunately, normalization of the IC content of our preparations could not be performed as PEG precipitates should be used fresh.

As a whole, the data presented in this work might impact our current understanding of SSc pathogenesis: SSc-ICs could provide an additional player in the complex interplay between autoimmunity, vascular damage and excessive fibroblast activation culminating in tissue fibrosis in the initiator phase of the disease. The relevance of SSc-ICs might account for the strong diagnostic and prognostic role scleroderma autoantibodies exert. Autoantibody production might be favored by environmental factors together with a predisposing genetic milieu, documented by the strong association with HLA assets and polymorphisms in TLRs and downstream mediators. Hopefully, further characterizing the pathogenic role of scleroderma autoantibodies could allow developing novel therapeutic strategies for a still barely treatable disease.

## Conclusion

This study shows that sera from scleroderma patients contain ICs and proposes for the first time the potential pathogenicity of SSc-ICs. Indeed, using skin fibroblasts from NHS as an in-vitro model, we observed that SSc-ICs can trigger proinflammatory and profibrotic mediators. These effects might be mediated by TLRs via interaction with nucleic acid fragments embedded in SSc-ICs. Our data suggest that SSc-ICs might be a novel player in the pathogenesis of scleroderma, fitting well with the diagnostic and prognostic role of SSc-specific autoantibodies.

## Abbreviations

ACA: Anti-centromeric protein antibodies
AFA: Anti-fibroblast antibodies
ANA: Anti-nuclear antibodies
anti-Th/To: Anti-Th/To antibodies
ARA: Anti-RNA polymerase III antibodies
ATA: Anti-DNA topoisomerase I antibodies
colIα1: CollagenIα1
DMEM: Dulbecco’s modified Eagle’s medium
EBV: Epstein–Barr virus
et-1: Endothelin-1
FBS: Fetal bovine serum
FcγR: Fcγ receptor
HBSS: Hank’s balanced salt solution
IC: Immune complex
IFN: Interferon
IL: Interleukin
LAL: Lymulus amoebocyte lysate
LPS: Lipopolysaccharide
MCP: Monocyte chemoattractant protein
MMP: Matrix metalloproteinase
NFκB: Nuclear factor kappa B
NHS: Normal healthy subjects
OD: Optical density
PT: PBS/0.05% Tween 20
p38MAPK: p38 mitogen activated kinase
PAPS: Primary anti-phospholipid syndrome
PEG: Polyethylene-glycol
pNFκB: Phosphorylated nuclear factor kappa B
Poly(I:C): Polyinosinic-polycytidylic acid
pp38MAPK: Phosphorylated p38MAPK
RNP: Ribonucleoprotein
RQ: Fold change
RT-PCR: Real-time PCR
SD: Standard deviation
SLE: Systemic lupus erythematosus
SSc: Systemic sclerosis
TGF: Tumor growth factor
TLR: Toll-like receptor
